# Public Health Approaches to Promote Health Equity for Individuals with Intellectual and Developmental Disabilities: A Systematic Review

**Published:** 2025-10-30

**Authors:** E Okoye, J Bronson, R Breland, A Omondi, M Shaw

**Affiliations:** 1. Department of Epidemiology & Biostatistics, College of Health Sciences, Jackson State University, Jackson, MS, USA; 2. Department of Health Policy & Management, College of Health Sciences, Jackson State University, Jackson, MS, USA; 3. Special Olympics Mississippi, Jackson, MS, USA; 4. Department of Behavioral & Environmental Health, College of Health Sciences, Jackson State University, Jackson, MS, USA

**Keywords:** Intellectual disability, developmental disability, health equity, public health, healthcare disparities, healthcare access, care coordination

## Abstract

**Background:**

Individuals with intellectual and developmental disabilities (IDD) experience persistent health disparities due to systemic barriers in healthcare access, limited provider training, and exclusion from public health research and interventions.

**Objective:**

To identify and synthesize public health strategies, interventions, and/or policies that effectively promote health equity among individuals with IDD.

**Methods:**

This systematic review followed PRISMA guidelines and included 33 peer-reviewed studies, qualitative, quantitative, and mixedmethods, published in English. Eligible studies focused on public health approaches to enhancing healthcare access and health outcomes for individuals with IDD. The methodological quality of the included studies was assessed using the Mixed Methods Appraisal Tool (MMAT), while the quality and strength of evidence were further evaluated using the GRADE approach. Data synthesis was conducted through thematic analysis.

**Results:**

Six key themes emerged: (i) training for healthcare providers, (ii) early intervention and preventive care, (iii) healthy lifestyle promotion, (iv) accessible and inclusive healthcare, (v) research and data collection, and (vi) care coordination. Each approach contributed to improved health outcomes and reduced disparities in healthcare access and quality for individuals with IDD.

**Conclusion:**

Promoting health equity for individuals with IDD requires coordinated, system-level public health strategies. Interventions must be inclusive, scalable, and responsive to the complex needs of this population. Greater investment in training, research, data collection, and inclusive policy design is crucial for achieving sustainable equity.

## Introduction

Intellectual and developmental disability (IDD) refers to a neurodevelopmental condition characterized by impaired adaptive functioning and an intelligence quotient of less than 70 [1]. It is a lifelong condition that impacts individuals in key areas of health, well-being, education, employment, community participation, and economic sustainability [2–5].

In the United States, approximately 7.4 million people had IDD in 2017, including approximately 5.3 million children and two million adults [6]. Among this population, those with severe intellectual disabilities have a standardized mortality ratio of 8.4 [7,8]. However, individuals with IDD have historically faced health disparities, including inadequate access to high-quality medical care, insufficient preparation of healthcare providers to meet their needs, a high prevalence of unmet social needs, and exclusion from public health research programs [9,10].

Individuals with IDD are at greater risk of health issues, including secondary diseases, co-morbidities, and lifestyle-related health problems, which could be mitigated with early primary care interventions [11]. Individuals with IDD face significant health disparities relative to the general population. While their life expectancy has improved over time, it still lags notably behind that of the broader population [7,8,12]. Additionally, extensive research indicates physical health inequities among the IDD population, including higher rates of chronic conditions, limited access to healthcare services, lower life expectancy, and higher rates of co-occurring diseases [13–20].

These disparities are compounded by communication difficulties, limited health literacy, and inadequate training of healthcare providers in disability-inclusive care [21]. Research reveals significant disparities in healthcare access, utilization, and health outcomes among adults with developmental disabilities [22]. Addressing these disparities and promoting health equity requires a comprehensive and targeted public health approach that acknowledges the unique needs of this population, addresses systemic barriers, and promotes inclusive and accessible healthcare [15,21].

To promote equitable access to healthcare services and improve health outcomes for individuals with IDD, adopting a public health approach provides a robust and holistic framework. This approach emphasizes preventive care, health promotion, and addressing the broader social determinants of health, while also considering the influence of policies and systems [23,24]. Key components of this framework include addressing social determinants, enhancing access to care, improving training for healthcare providers, promoting collaboration between health and disability service sectors, and bolstering advocacy initiatives [21,23].

This study examines public health approaches, strategies, interventions, and/or policies that promote health equity for individuals with intellectual and developmental disabilities. By systematically reviewing existing strategies, interventions, and policies, the study identified evidence-based practices that effectively reduce healthcare disparities and improve health outcomes within this population. The findings will inform public health initiatives and guide policymakers in developing targeted, inclusive, and scalable programs and interventions that enhance access to care, preventive services, and overall health promotion for individuals with IDD.

## Methods

### Protocol

The Preferred Reporting Items for Systematic Reviews and Meta-Analyses (PRISMA) guideline (Liberati et al., 2009) was used to guide the systematic review. The protocol for this review was registered in the International Prospective Register of Systematic Reviews (PROSPERO; registration number CRD420251070037). The stages followed to perform the systematic review were based on (a) defining the appropriate keywords, (b) searching databases to find and select articles, (c) critical evaluation of the studies, (d) data selection and analysis, and (e) presentation and interpretation of results.

### Eligibility Criteria

This systematic review included original, peer-reviewed qualitative, quantitative, and mixed-method studies that examined public health approaches to promoting health equity among individuals with IDD.

#### Inclusion Criteria

Eligible studies were English-language, peer-reviewed journal articles that focused on individuals with intellectual and developmental disabilities (IDD) as the primary population. Both primary and secondary research studies were included if they addressed health disparities, health inequities, or public health approaches relevant to the IDD population. Studies involving individuals of all age groups were eligible, and only full-text articles were considered. There were no restrictions based on geographic location or country of study.

#### Inclusion Criteria

The exclusion criteria included non-English publications, literature reviews, systematic reviews, book chapters, and conference proceedings. Studies were also excluded if they focused on populations other than individuals with intellectual and developmental disabilities (IDD) or did not examine public health approaches aimed at promoting health equity for the IDD population.

### Search Strategy

The researchers (EO & JB) conducted a comprehensive literature search on June 11, 2023, and June 3, 2025, across six electronic databases: PubMed, CINAHL, MEDLINE, ERIC, PsycINFO, and Health Source. The search focused on peer-reviewed journal articles published in English. It utilized combinations of keywords such as “intellectual and developmental disabilities,” “health disparities,” “health equity,” “public health approaches,” “health promotion”, “accessible healthcare,” and “interventions.” Additionally, manual searches of reference lists from included articles were performed to capture any relevant studies missed in the initial search. The study selection process followed PRISMA (Preferred Reporting Items for Systematic Reviews and Meta-Analyses) guidelines to ensure transparency and rigor in article identification, screening, and inclusion. Medical Subject Headings (MeSH) terms were used exclusively in PubMed to enhance the precision of the search strategy, while keyword-based searches were employed for the remaining databases.

The screening was conducted in two phases: an initial review of titles and abstracts, followed by a full-text screening. The inclusion criteria were applied at both stages. A total of 148 articles were selected for full-text review. Two independent reviewers conducted the full-text screening, with any discrepancies resolved through weekly group discussions. After excluding 115 articles that did not meet the criteria, 33 were eligible and included in the final systematic review.

### Study Screening Process

**Figure 1:**
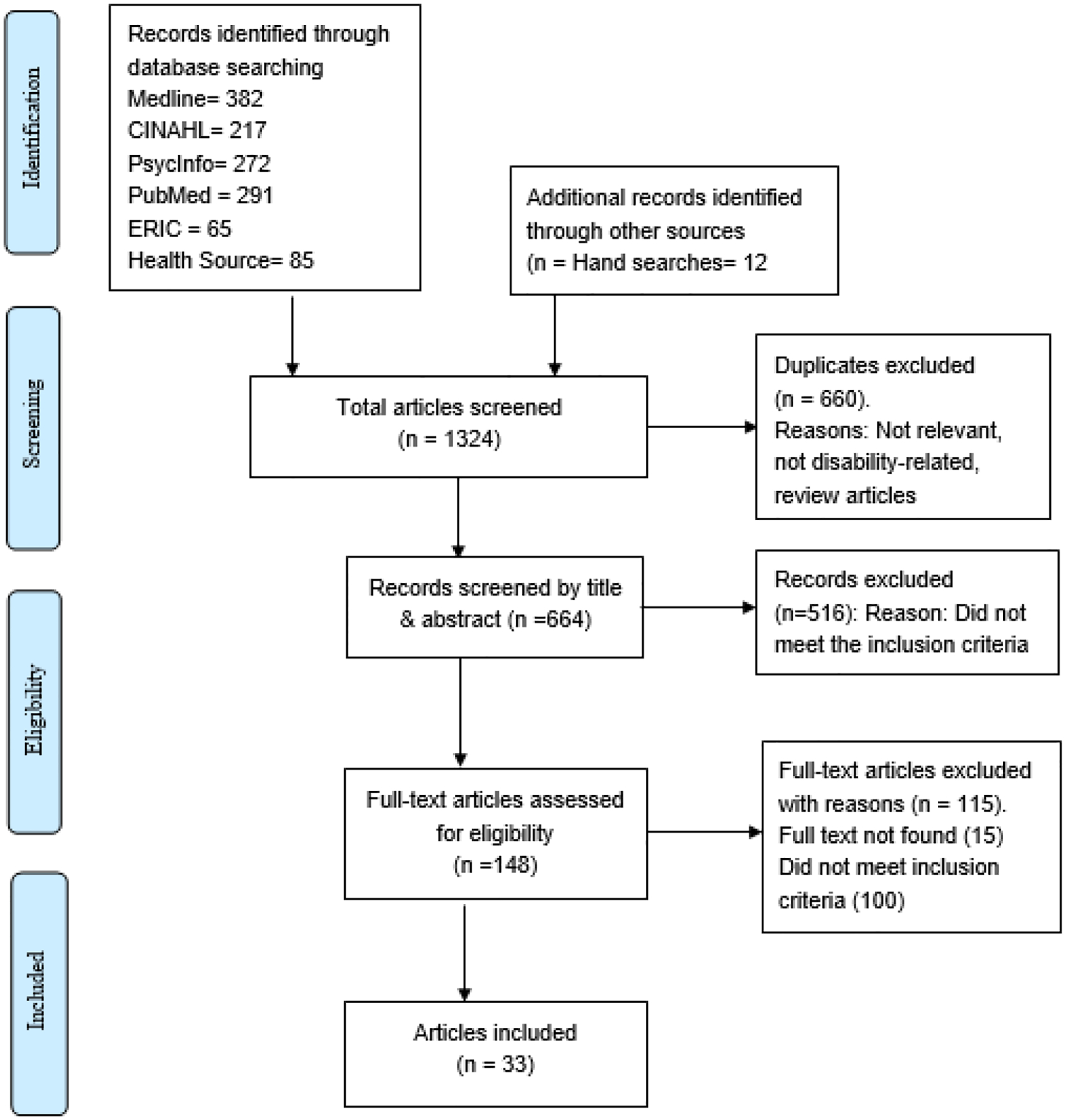
PRISMA flow chart

The researchers (EO & JB) independently the searched across selected databases using the same keywords. This initial search yielded 1,312 articles, with an additional 12 articles identified through manual hand-searching. The PRISMA flowchart in Figure 1 [25] outlines the search process, showing that a total of 1,324 articles were identified. After removing 660 duplicates, 664 articles remained for screening.

### Quality Assessment

The researchers (EO & JB) independently assessed the quality of each included study using the Mixed Methods Appraisal Tool (MMAT) developed by Hong et al. [26]. This tool was chosen for its ability to systematically evaluate diverse study designs, including qualitative studies, quantitative descriptive and non-randomized studies, randomized controlled trials, and mixed methods research. During the critical appraisal process, researchers evaluated how well each study’s design aligned with its stated objectives, providing essential context for interpreting findings. Each study was assessed using the MMAT’s specific domains, and a quality rating was assigned based on the proportion of criteria met. The following scale was used: 100% (***** = high quality), 80% (**** = good), 60% (*** = satisfactory), 20% (** = poor), and <20% (* = very poor). To ensure consistency, independent assessments were compared, and consensus was reached through discussion. Any uncertainties were resolved collaboratively among the research team. Notably, study quality did not serve as an exclusion criterion. Instead, ratings were used to interpret the reliability of evidence during the synthesis. This approach allowed the inclusion of a broad range of perspectives while acknowledging methodological variation across studies.

In addition to MMAT, the GRADE (Grading of Recommendations Assessment, Development, and Evaluation) approach was used to assess overall strength and certainty of evidence across key outcomes. GRADE evaluates evidence based on study limitations, consistency of findings, precision, directness, and publication bias. This structured framework provided a transparent way to rate confidence in effect estimates across the body of evidence. The GRADEpro software was used to generate the Summary of Findings table [27].

### Data Extraction

Data was extracted using a structured form developed and piloted by the research team. Key information included authors, publication year, article type, country, study design, and quality rating. The thematic analysis [28] approach was used to identify recurring patterns and insights across the studies. The researchers (EO & JB) independently generated preliminary themes, which were reviewed and refined through discussion to reach consensus. The final themes were narratively synthesized to present a cohesive summary of findings.

## Results

Table 1 summarizes the information extracted from each included study, detailing the author(s), key findings, year of publication, study design, quality appraisal rating, and country of study. A total of thirty-three (33) studies met the eligibility criteria and were reviewed. Of these, 17 employed quantitative methods, 12 used qualitative approaches, and 4 utilized mixed methods designs. In terms of publication timelines, 16 studies were published between 2004 and 2016, while the remaining 17 were published between 2018 and 2023. The quality and strength of evidence of the articles included were further evaluated using the Grading of Recommendations Assessment, Development and Evaluation (GRADE) approach. The strength and consistency of the evidence of included articles across the key outcomes were assessed using a GRADE summary of findings table (Table 2). This table synthesizes the effects of public health strategies on six outcome areas relevant to promoting health equity for individuals with IDD. The outcomes include provider knowledge and communication, utilization of preventive healthcare services, physical health and wellness, increased access and care experience, inclusion of individuals with IDD in health data and research, and continuity of care.

The majority of the articles included in this study were conducted in the following countries: the United States (n = 14), the United Kingdom (n = 7), Australia (n = 3), the Netherlands (n = 2), Germany (n = 2), Ireland (n = 2), Canada (n = 1), Sweden (n = 1), and Taiwan (n = 1). Primarily, all included studies focused on exploring the perspectives and experiences of individuals with IDD.

Of the 33 studies analyzed, 26 (79%) were rated as high quality, 5 (15%) as good quality, and 2 (6%) as satisfactory based on the MMAT appraisal. However, no studies were excluded based solely on their quality appraisal scores. This decision was based on the understanding that the MMAT primarily assesses the quality of study reporting rather than methodological rigor or the substantive relevance of the research. By including all studies regardless of appraisal results, the review provided a more comprehensive synthesis and derived valuable insights from a broad range of evidence [29]. Thematic analysis conducted by the researchers (EO & JB) identified six recurring themes that represent the public health approaches, strategies, and/or interventions employed to promote health equity among individuals with IDD, as outlined below.

### Training for Health Providers

Of the 33 studies reviewed, three (3) articles (9%) specifically highlighted the significance of training healthcare providers and stakeholders to enhance access to care for individuals with IDD. These studies emphasized that targeted education and professional development (training) are essential for equipping healthcare providers with the knowledge and skills needed to understand and address the distinct health needs and challenges of individuals with IDD [30–32]. Furthermore, Overwijk et al. [32] reported that healthcare providers who received adequate training are better equipped to understand and communicate with individuals with moderate to profound IDD, making them more effective in supporting them to adopt and sustain healthy lifestyle behaviors.

### Early Intervention & Preventive Care

Out of the 33 studies examined, five (15%) highlighted early intervention and preventive care as critical strategies for improving health outcomes among individuals with IDD. These studies reported that access to primary healthcare services can significantly enhance healthcare utilization and help reduce disparities within this population [33–37]. Additionally, three studies reported the effectiveness of health screenings and annual health checks as key interventions for early disease detection among individuals with IDD, contributing to the reduction of health disparities [33–36]. Two other studies identified primary care services [37] and in-home preventive care delivered by advanced practice nurses [35] as effective approaches to enhancing healthcare outcomes for this population.

### Healthy Lifestyle Promotion

Among the 33 studies analyzed, six (18%) highlighted the effectiveness of healthy lifestyle interventions as public health strategies/approaches for advancing health equity among individuals with IDD [38–43]. Two (2) of these studies specifically examined the impact of the Special Olympics Fit 5 program and other community-based health promotion initiatives tailored for Special Olympics athletes [39,41]. These interventions were associated with improved perceived health, reduced body weight, and decreased barriers to physical activity among individuals with IDD. Additionally, four (4) studies examined broader healthy lifestyle change programs on individuals with IDD and reported positive outcomes, including enhanced overall lifestyle behaviors and well-being in this population [38,40,42,43].

### Accessible & Inclusive Healthcare

Among the 33 studies analyzed, two (6%) studies [44,45] reported the effectiveness of accessible and inclusive health strategies as public health approaches to advancing health equity for individuals with IDD. Finlayson et al. [45] reported positive outcomes when individuals with IDD participated in interventions such as DXA (Dual-Energy X-ray Absorptiometry) and BMD (Bone Mineral Density) screenings, provided with reasonable adjustments. These interventions contributed to reducing health disparities commonly observed in this population, showing the importance of tailored healthcare interventions that address their specific needs. In contrast, McConkey et al. [42] focused on identifying inclusive health actions and system-level indicators that, if adopted, could improve the overall inclusivity of health systems for individuals with IDD.

### Research & Data Collection

Of the 33 studies reviewed, four (12%) reported the critical role of research and data in advancing health equity for individuals with IDD [20,41,46,48]. Tyler Jr et al. [20] used electronic health records to assess the prevalence of chronic diseases among individuals with IDD and found notable discrepancies when compared to earlier reports, revealing significant gaps in existing health surveillance systems. Bains & Turnbull [47] conducted behavior-change theory-based interviews among people with IDD and applied an iterative data collection approach, which enhanced participant engagement and inclusion of individuals with IDD in the research process. Wilkinson et al. [48] found that using a comprehensive dataset for health surveillance among individuals with IDD revealed inconsistent representation, with adults living with their families being better represented than those living independently or in other settings. Additionally, McDonald [46] reported that actively involving individuals with IDD as direct research participants fosters first-person decision-making and supports their meaningful engagement in the research process.

### Care Coordination

Among the 33 studies reviewed, two (6%) reported care coordination as an essential public health strategy for advancing health equity among individuals with IDD [49,50]. Gadd [49] reported the value of person-centered respite support in enhancing the overall well-being of individuals with IDD. Similarly, Ruiz et al. [50] reported that the implementation of care coordination models led to improved health outcomes, reinforcing the importance of integrated and individualized care approaches for this population.

## Discussion

This systematic review indicates the key public health strategies/approaches that promote health equity for individuals with IDD. Findings are discussed regarding existing literature, focusing on identifying effective interventions and system-level approaches that promote healthcare access and health outcomes for individuals with IDD. While numerous programs have been developed to support individuals with IDD, this review identified only six categories of interventions that demonstrated measurable effectiveness in advancing health equity. These include: (i) training for healthcare providers, (ii) early intervention and preventive care, (iii) healthy lifestyle promotion, (iv) accessible and inclusive healthcare, (v) research and data collection, and (vi) care coordination.

Each of these themes reflects critical areas where public health efforts can reduce disparities and create more equitable healthcare systems. The following discussion interprets the significance of these findings, explores gaps in implementation and research, and offers implications for future practice and policy.

Results indicate that training healthcare providers plays an important role in advancing health equity for individuals with IDD. This aligns with previous research demonstrating that comprehensive training programs can significantly improve healthcare professionals’ knowledge, skills, and attitudes, thereby enhancing the quality of care for individuals with IDD [51,52]. Additionally, Rubinelli et al. [53] reported that effective communication, rooted in a provider’s understanding of IDD-specific needs, is essential for achieving positive health outcomes. Effective communication skills are essential for healthcare providers to build trust, identify the unique needs of individuals with IDD, and provide person-centered care. Enhancing provider communication not only fosters better clinical interactions but also contributes to reducing disparities by promoting more equitable and respectful treatment. Training interventions that focus on disability awareness and person-centered approaches have been shown to improve providers’ ability to communicate, understand specific health concerns, and respect the autonomy and rights of individuals with IDD. These competencies are foundational to advancing health equity and improving the overall quality of care for this population.

Early intervention and preventive care play a crucial role in improving the health and long-term well-being of individuals with IDD. A similar finding by Rosenbaum et al. (2012) reported that early identification and targeted interventions led to improved developmental outcomes and reduced risk of long-term disabilities. According to Smith et al. [54], preventive care measures, such as routine health screenings, risk assessments, and timely referrals, are essential for the early detection of health risks and the prevention of disease progression among individuals with IDD. Additionally, promoting optimal early childhood development yields long-term benefits that extend beyond health, including improved educational attainment, increased productivity in adulthood, and enhanced population health outcomes [55,56]. These findings highlight the importance of embedding early intervention and preventive care into health systems, particularly for children with IDD and their families, as a strategic approach to advancing health equity.

Implementing healthy lifestyle interventions is critical for promoting health equity among individuals with IDD. Although only 18% of the reviewed studies addressed healthy lifestyle interventions, findings consistently support their value in promoting health equity for individuals with IDD. Studies consistently show programs such as structured exercise, nutrition education, and community-based wellness initiatives have led to improved physical health, well-being, and reduced barriers to participation [57–59]. Additionally, walking programs have also shown positive outcomes in supporting adults with IDD [59,60]. Despite these benefits, lifestyle interventions remain underutilized and often lack long-term sustainability. Many are not integrated into public health systems and fail to involve individuals with IDD in their design, limiting accessibility and impact. To be effective and equitable, future efforts should focus on embedding inclusive health promotion into routine services, fostering community partnerships, and ensuring long-term support and evaluation.

Accessible and inclusive healthcare is essential to achieving equity for individuals with IDD. It highlights the need for structural changes and policy interventions to create more inclusive healthcare environments that address the specific barriers faced by individuals with IDD. This is consistent with Selick et al. [34], who reported that reinforcing structural and policy-level changes to address persistent barriers, including communication challenges and inadequate provider training, is essential for ensuring timely and appropriate care. Implementing disability-friendly practices, such as simplified communication tools and supportive environments, can enhance care experiences and patient satisfaction. Research also indicates that empowering individuals with IDD to participate actively in their care enhances decision-making and improves health outcomes (Sullivan & Heng, 2018). Ultimately, improving provider knowledge and attitudes remains a key factor in ensuring equitable healthcare access and quality[16].

Research and data collection play a role in identifying health disparities and informing targeted interventions for individuals with IDD. However, their underrepresentation in health research and surveillance remains a critical barrier to equity. Only 12% of the reviewed studies addressed research and data collection, indicating a major gap in disability-specific surveillance. Individuals with IDD are often underrepresented in health data due to exclusionary research practices and inaccessible methodologies [20,48]. This lack of data perpetuates inequities by limiting evidence-based policy and service planning. Additionally, studies by McDonald [46] and Bains & Turnbull [47] show the value of participatory research methods that actively engage individuals with IDD. To advance equity, public health systems must invest in inclusive research practices and collect disaggregated data that reflects the full diversity of the IDD population. This finding is consistent with Havercamp et al. [61], who reported that data collection through epidemiological studies, surveys, and health assessments provides valuable insights into healthcare access, utilization, services, and outcomes for individuals with IDD. These insights are essential for developing targeted strategies and policies that promote equity and improve health outcomes for these individuals.

Care coordination, although addressed in only 6% of the reviewed studies, plays a critical role in improving health outcomes for individuals with IDD. Navigating complex healthcare systems and fragmented services can be particularly challenging for this population [62]. Effective care coordination facilitates access, reduces duplication, and promotes continuity of care. It has been linked to improved health outcomes, medication management, and overall well-being [63]. Person-centered models, such as individualized respite care and structured coordination frameworks, further enhance continuity and reduce service fragmentation [49,50]. However, despite these benefits, implementation remains limited due to funding constraints, siloed systems, and workforce shortages. For individuals with IDD who frequently engage with multiple health and social service sectors, coordinated care is essential to prevent delays, duplications, and gaps in treatment. Embedding care coordination into primary and public health systems is vital for delivering equitable, efficient, and person-centered care to individuals with IDD.

According to the GRADE Summary of Findings Table (Table 2), the strongest evidence was observed for the outcome utilization of preventive healthcare services, supported by five randomized controlled trials (RCTs) and rated as “high-certainty evidence”. Moderate-certainty evidence was found for improved care coordination and health screenings uptake, backed by mixed-method and non-randomized studies. Outcomes such as health promotion engagement and policy and advocacy improvements were supported by lower-quality study designs and were rated “low to very low certainty ratings”, primarily due to methodological limitations, inconsistency, and indirectness. Overall, the findings suggest a positive trend toward improving health equity for individuals with IDD, though further high-quality research is warranted [64–67].

## Conclusion

This study revealed the critical role of public health approaches in promoting health equity for individuals with IDD. The review of thirty-three studies reveals that interventions such as training, research and data collection, early intervention, care coordination, accessible healthcare, and healthy lifestyle promotion are vital in advancing health equity for this population. By implementing these approaches, we can effectively address health disparities and strive for equitable healthcare for individuals with IDD. Sustained efforts to integrate and prioritize public health strategies are essential to achieving optimal health outcomes and enhancing the overall well-being of individuals with IDD.

### Implications for Practice and Policy

Despite promising evidence, most interventions were small-scale, lacked long-term evaluation, and were rarely integrated into broader healthcare or policy frameworks. The consistent underrepresentation of individuals with IDD in research and program design limits the scalability of many initiatives. There is a pressing need for disability-inclusive public health policies that embed accessible care, coordinated services, workforce training, and disability-disaggregated data collection into core health system functions. Individuals with IDD must be actively engaged in shaping these systems, not merely counted.

### Future Research

Future research should focus on large-scale, methodologically rigorous studies that examine the effectiveness, sustainability, and equity impact of public health interventions targeting the IDD population. Emphasis should be placed on participatory and community-engaged research designs, where individuals with IDD contribute meaningfully to defining priorities, outcomes, and approaches. Such inclusive methods are necessary to ensure interventions are relevant, scalable, and grounded in the lived experiences of those most affected.

## Limitations

This review has several limitations that warrant consideration. Despite comprehensive efforts to capture all relevant literature using appropriate search terms and multiple databases, the possibility remains that some eligible studies were inadvertently excluded. Additionally, the review was limited to peer-reviewed, published journal articles; unpublished data and grey literature were not included, which may have introduced publication bias.

Another notable limitation is the predominant focus of the included studies on individuals with mild intellectual and developmental disabilities (IDD). This restricts the generalizability of the findings to those with more severe forms of IDD, for whom health disparities may be more pronounced and interventions may require different approaches. Future studies should aim to assess the effectiveness of public health strategies across a broader spectrum of disability severity.

Furthermore, many of the interventions reviewed relied heavily on written or verbal communication, which may pose accessibility challenges for individuals with communication or cognitive impairments. Alternative formats or increased caregiver involvement may be necessary to ensure meaningful engagement and equitable outcomes for all participants.

Lastly, although the review focused on individuals with IDD, the findings highlight the potential for adapting and extending these public health interventions to individuals with other types of disabilities. Broader inclusion and tailored approaches are needed to address the diverse and intersecting needs within the wider disability community.

## Recommendation

Individuals with IDD have diverse needs that span both physical and mental health domains. To address these effectively, health services and interventions must be tailored specifically to this population. Current systems often lack the flexibility or specialization required to adequately meet the unique health needs of individuals with IDD. Therefore, implementing specialized, person-centered approaches is critical to ensuring equitable access to care and improving overall health outcomes. These approaches should be embedded within broader health systems and supported by informed policies, trained professionals, and inclusive program designs that prioritize the voices and experiences of individuals with IDD.

## Figures and Tables

**Table 1:** Characteristics of included studies

Authors (Ref)	Major findings	Study type	Quality appraisal	Country of study
O’Donovan et al., 2018	Irish health policies lacked commitment to core human rights principles tailored to the needs of people with disabilities, including those with intellectual disabilities, despite general equity goals.	Qualitative	***	Ireland
McDonald, 2022	Including adults with intellectual disabilities as direct respondents, promoting first-person decision-making through accessibility, trust, respect, and engagement.	Qualitative	*****	USA
McCarty et al., 2020	Health and wellness programs for individuals with IDD received positive feedback, with continued participation anticipation and key recommendations identified.	Qualitative	*****	USA
Durbin et al., 2016	Health checks for adults with IDD were successfully and sustainably implemented at two Canadian primary care clinics, though outcomes differed between sites.	Qualitative	*****	CANADA
Wilkinson et al., 2014	The state database and hospital CDW were compared for IDD health surveillance. The state database showed inconsistent representation, with better coverage for adults in supported residences than for those living independently or with family.	Qualitative	****	USA
Tyler Jr. et al., 2010	EHR adoption revealed discrepancies between actual chronic disease prevalence in individuals with IDD and prior reports.	Qualitative	*****	USA
Perry et al., 2014	Participants reported generally positive experiences with primary care and health checks.	Qualitative	*****	UK
Bains, & Turnbull., 2022	Adults with intellectual disabilities successfully participated in a community-based, staged recruitment and iterative data collection process informed by behavior-change theory.	Qualitative	*****	UK
Finlayson et al., 2018	With reasonable adjustments, adults with intellectual disabilities, regardless of severity, can successfully complete DXA and BMD screenings. Scaling up these adjustments may reduce healthcare inequalities.	Descriptive Study	*****	UK
Bishop et al., 2023	Most health research excludes people with intellectual disabilities due to design, consent, resource, and training barriers. Ethics committees often exclude them to ‘protect’ them, but both researchers and people with IDs call for more inclusive, co-produced research.	Qualitative	*****	UK
Brandi et al., 2021	The Fit 5 program led to significant improvements in resting systolic and diastolic blood pressure among individuals with IDD compared to controls.	Non-Randomized study	****	USA
Iacono & Sutherland, 2004	The study reveals the value of health screenings for individuals with IDD, who showed higher engagement in urine tests, X-rays, and influenza vaccinations compared to the general population.	Descriptive Study	*****	Australia
Bartkowski et al., 2018	Clinician improvement programs (DETECT seminars and IDD-X) showed positive outcomes in treating patients with IDD, with high success across multiple evaluation measures.	Descriptive Study	*****	USA
McConkey, 2020	The findings show that strong support exists for inclusive health systems for individuals with IDD, though implementation remains a significant challenge across different settings.	Descriptive Study	****	USA
Hahn & Aronow, 2005	The implementation of in-home preventive Advanced Practice Nurse (APN) interventions promotes healthy aging and reduces health disparities in individuals with IDD.	Descriptive Study	****	USA
Buszewicz, 2014	Targeted annual health checks in primary care may reduce health inequities for individuals with IDD.	Non-Randomized study	*****	UK
Gadd, 2020	Person-centered respite supports significantly enhanced well-being and flourishing for individuals with intellectual disabilities.	Qualitative	*****	Ireland
Overwijk et al., 2022	A theory-based training program for direct support professionals effectively promoted healthy lifestyles in individuals with moderate to profound IDD.	Mixed Method	*****	Netherlands
Marks et al., 2010	Study shows that community-based health promotion programs for Special Olympics athletes yield positive psychosocial and health benefits, including improved perceived health, reduced body weight, and increased fiber intake.	Mixed Method	****	USA
Amir et al., 2022	Results reveal the need for systematic obstetric care training for women with IDD. Improved training can reduce health inequities, enhance care quality, and improve pregnancy outcomes.	Mixed Method	*****	USA
Ruiz et al., 2019	Care coordination models effectively addressed key contextual factors, including residential settings, health disparities, and the diverse needs of individuals with IDD.	Randomized-Controlled Trial	*****	USA
Lennox et al., 2016	School-based health advocacy packages for adolescents with IDD successfully increased healthcare engagement in the community.	Randomized-Controlled Trial	*****	Australia
Wu et al., 2010	Healthy fitness programs significantly benefited individuals with IDD, leading to notable weight loss and improved BMI scores within six months.	Descriptive Study	*****	Taiwan
Horner-Johnson et al., 2011	Healthy lifestyle interventions for individuals with IDD led to increased healthy behavior scores.	Descriptive Study	*****	USA
Alicia et al., 2009	A study on healthy lifestyle changes in adults with developmental disabilities showed improved behaviors, successful weight loss, and enhanced community capacity.	Randomized-Controlled Trial	***	USA

**Table 2:** Grade Summary of Findings: Effectiveness of public health strategies in promoting health equity for individuals with IDD

Should public health strategies be used to achieve health equity for individuals with intellectual and developmental disabilities?					
**Patient or population:** Achieving health equity for individuals with intellectual and developmental disabilities **Setting:** Systematic Review **Intervention:** Public health strategies **Comparison:** Nothing					
				Anticipated absolute effects	
	Risk with nothing № of participants (studies) Follow-up	Risk difference with using public health strategies Certainty of the evidence (GRADE)			
Outcomes			Relative effect (95% CI)		
**Outcome:** Improved provider knowledge, communication, and patient interactions **Assessed with:** Knowledge tests, self-reported provider confidence, communication behavior scales **Follow-up:** mean 6 months	41 (3 non-randomized studies)	⨁◯◯◯ Very low	**RR 0.96** (0.69 to 1.32)	**Low**	
				0 per 100	**0 fewer per 100** (0 fewer to 0 fewer)
**Outcome:** Utilization of preventive healthcare services **Assessed with**: Rates of screenings, health checks, medical records, primary care practice records **Follow-up**: range 6 months to 2 years	19206 (5 RCTs)	⨁⨁⨁⨁ High	**RR 2.63** (2.46 to 2.83)	10 per 100	**16 more per 100** (14 more to 17 more)
**Outcome:** Physical health & wellness **Assessed with**: Health Promoting Lifestyle Profile II, access to care, self-reported nutrition **Follow-up**: range 2 weeks to 10 months	706 (6 RCTs)	⨁⨁⨁⨁ High	not estimable	0 per 100	**0 fewer per 100** (0 fewer to 0 fewer)
**Outcome:** Access and care experience **Assessed with**: Patient satisfaction surveys, service utilization data, and no-show rates **Follow-up**: mean 6 months	330 (2 non-randomized studies)	⨁⨁◯◯ Low	not estimable	0 per 100	**0 fewer per 100** (0 fewer to 0 fewer)
**Outcome:** Inclusive in health data and research **Assessed with**: Inclusion rate of individuals with IDD in research samples, availability of disaggregated data by disability status, involvement of individuals with IDD in study design, data collection, or analysis **Follow-up**: No reported	3801 (5 non-randomized studies)	⨁⨁◯◯ Low	not estimable	0 per 100	**0 fewer per 100** (0 fewer to 0 fewer)
**Outcome:** Improved continuity of care & care navigation through coordination models **Assessed with**: Care plan documentation, follow-up rate, coordination records **Follow-up**: 4 years	100 (2 non-randomized studies)	⨁⨁◯◯ Low	**RR 0.41** (0.26 to 0.64)	74 per 100	**44 fewer per 100** (55 fewer to 27 fewer)
*The risk in the intervention group (and its 95% confidence interval) is based on the assumed risk in the comparison group and the **relative effect** of the intervention (and its 95% CI). **CI:** confidence interval; **RR:** risk ratio **GRADE Working Group grades of evidence** **High certainty:** We are very confident that the true effect lies close to that of the estimate of the effect. **Moderate certainty:** We are moderately confident in the effect estimate: the true effect is likely to be close to the estimate of the effect, but there is a possibility that it is substantially different. **Low certainty:** our confidence in the effect estimate is limited; the true effect may be substantially different from the estimate of the effect. **Very low certainty:** we have very little confidence in the effect estimate; the true effect is likely to be substantially different from the estimate of effect.					
